# Shared HLA Class II in Six Autoimmune Diseases in Latin America: A Meta-Analysis

**DOI:** 10.1155/2012/569728

**Published:** 2012-04-19

**Authors:** Paola Cruz-Tapias, Oscar M. Pérez-Fernández, Adriana Rojas-Villarraga, Alberto Rodríguez-Rodríguez, María-Teresa Arango, Juan-Manuel Anaya

**Affiliations:** ^1^Center for Autoimmune Diseases Research (CREA), School of Medicine and Health Sciences, Universidad del Rosario, Carrera 24 No. 63C-69, Bogotá, Colombia; ^2^Doctoral Program in Biomedical Sciences, Universidad del Rosario, Bogotá, Colombia

## Abstract

The prevalence and genetic susceptibility of autoimmune diseases (ADs) may vary depending on latitudinal gradient and ethnicity. The aims of this study were to identify common human leukocyte antigen (HLA) class II alleles that contribute to susceptibility to six ADs in Latin Americans through a meta-analysis and to review additional clinical, immunological, and genetic characteristics of those ADs sharing HLA alleles. DRB1^*∗*^03:01 (OR: 4.04; 95%CI: 1.41–11.53) was found to be a risk factor for systemic lupus erythematosus (SLE), Sjögren's syndrome (SS), and type 1 diabetes mellitus (T1D). DRB1^*∗*^04:05 (OR: 4.64; 95%CI: 2.14–10.05) influences autoimmune hepatitis (AIH), rheumatoid arthritis (RA), and T1D; DRB1^*∗*^04:01 (OR: 3.86; 95%CI: 2.32–6.42) is a susceptibility factor for RA and T1D. Opposite associations were found between multiple sclerosis (MS) and T1D. DQB1^*∗*^06:02 and DRB1^*∗*^15 alleles were risk factors for MS but protective factors for T1D. Likewise, DQB1^*∗*^06:03 allele was a risk factor for AIH but a protective one for T1D. Several common autoantibodies and clinical associations as well as additional shared genes have been reported in these ADs, which are reviewed herein. These results indicate that in Latin Americans ADs share major loci and immune characteristics.

## 1. Introduction

Autoimmune diseases (ADs) are chronic conditions initiated by the loss of immunological tolerance to self-antigens. They are a heterogeneous group of disorders that affect specific target organs or multiple organ systems [1]. Almost all ADs disproportionately affect middle-aged women and are among the leading causes of death for this group of patients [2]. The etiology of ADs is unknown, but these complex diseases are known to feature genetic and environmental factors in their development [1, 3]. Although they exhibit contrasting epidemiological features and clinical manifestations, there is evidence that ADs share similar immunogenetic mechanisms [4].

Three related lines of evidence sustain the common origin for ADs. First, clinical evidence highlights the cooccurrence of distinct ADs within an individual (i.e., polyautoimmunity) and within members of a nuclear family (i.e., familial autoimmunity). Second, physiopathologic evidence indicates that the pathologic mechanisms may be similar among ADs. Third, genetic evidence shows that autoimmune phenotypes might represent pleiotropic outcomes of the interaction of nonspecific disease genes [5].

The study of HLA, which carries the major genetic influence on susceptibility to ADs, will allow us to understand its common or specific influence on these diseases and to identify genetic prediction markers. The large and diverse population of Latin America (LA) is a powerful resource for elucidating the genetic basis of complex traits due to its admixture [6]. Modern day LA resulted from the encounter of Europeans with the indigenous people of the Americas in 1492, followed by waves of migration from Europe and Africa. As a result, the genomic structure of present day Latin Americans is determined by both the genetic structure of the founding populations and the numbers of migrants from these different populations [7]. Analysis of multiple Latino populations in gene association studies could also strengthen the potential associations as well as provide opportunities for examining gene-environment and gene-gene interactions [8].

The purpose of this paper was to estimate the common effect size of HLA class II on ADs across LA populations through a meta-analysis and to evaluate the additional characteristics (i.e., other genes, autoantibodies, and clinical characteristics) of genetically associated ADs in Latin America.

## 2. Materials and Methods

### 2.1. Study Selection

Five meta-analyses of HLA class II polymorphisms in LA patients with ADs published from 2007 to 2010 by our group were included [9–13]. The ADs included were rheumatoid arthritis (RA), systemic lupus erythematosus (SLE), autoimmune hepatitis (AIH), multiple sclerosis (MS), and type 1 diabetes (T1D). In addition, the results from the only study of Sjögren's syndrome (SS) reported on the LA population were included [14]. Briefly, the strategies to search for, select, and analyze the studies used for each meta-analysis are mentioned hereinafter.

In all of the cases, a systematic review of the electronic databases (MEDLINE, PubMed, SciELO, BIREME, EMBASE, Cochrane, and LILACS) was done independently by two experts. The searches only included publications on HLA-Class II alleles and susceptibility to ADs in LA published in any of these three languages: Spanish, English, or Portuguese. All of the search strategies included MeSH terms: “HLA DR/DQ antigens” and “Major Histocompatibily Complex”. However, other major topics were used depending on the specific AD: “Arthritis, Rheumatoid”, “Lupus Erythematosus, Systemic”, “Hepatitis, Autoimmune”, “Hepatitis, Chronic”, “Multiple Sclerosis”, “Type 1 Diabetes”, or “Autoimmune Diabetes”.

The inclusion criteria were the following: (1) AD diagnosis established using international validated criteria for RA [15], for SLE [16], for AIH [17, 18], for MS [19, 20], and for T1D [21, 22]; (2) case-control design of the study; (3) publication of sufficient information to calculate odds ratios (ORs); (4) a focus on a well-defined LA population; (5) use of molecular techniques to determine HLA polymorphisms (i.e., allele-specific oligonucleotides—ASO, polymerase chain reaction with sequence-specific primers—PCR/SSP, restriction fragment length polymorphism—RFLP, specific oligonucleotide probes—SOP, or sequence-specific oligonucleotide probes—SSOP); and (6) manuscript's publication in a peer-reviewed journal as a full paper. Summaries or abstracts were not accepted.

Data were analyzed using the Comprehensive Meta-Analysis version 2 program (Biostat, Englewood, NJ, 2004). For each polymorphism group, the effect summary odds ratio (OR) and 95% confidence interval (CI) were obtained by the random effect model. The systematic review and meta-analysis were done following the PRISMA guidelines and the respective checklist completion [23].

### 2.2. Meta-Analysis

 Calculations were carried out for each HLA-DR and HLA-DQ allele using low or high resolution based on information available in each meta-analysis. The final pooled OR was done by weighing individual OR by the inverse of their variance. For each allele, the final effect OR and 95%CI were obtained by means of a random model. This model was used because of the assumption that there is a distribution of true effect sizes rather than one true effect, assigning a more balanced weight to each study. It was also used because all the studies were considered to be functionally unequal. Values less than 1.0 suggest a protective effect while values greater than 1.0 suggest a risk for each AD. Heterogeneity was calculated by means of Cochran's (*Q*) and Higgins's (*I* ^2^) tests. The *I* ^2^ test measures the degree of inconsistency in the studies by calculating the percentage of total variation across studies due to heterogeneity rather than chance and was expressed as a ratio with a range of 0% to 100%. A qualitative classification of low, moderate, and high were assigned to *I* ^2^ values of 25%, 50%, and 75%, respectively. A significant *Q*-statistic (*P* < 0.10) indicated heterogeneity across studies. Publication bias was determined using Funnel plots, Egger's regression asymmetry tests, and sensitivity analysis. Data were analyzed by using Comprehensive Meta-Analysis version 2 program.

### 2.3. Literature Review

**Figure 1:**
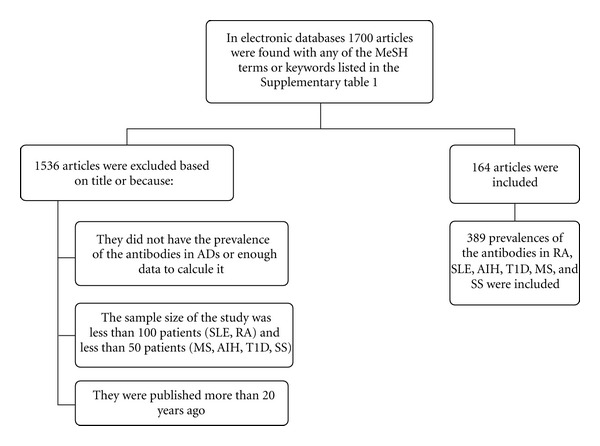
Flow chart of the systematic literature review.

An updated systematic literature review was done following the PRISMA guidelines [23] for the prevalence of autoantibodies in RA, SLE, AIH, T1D, SS, and MS (Figure 1). Publications were identified through a systematic search done in Pubmed. The inclusion criteria were the following: (1) studies in humans, (2) restricted by title, (3) articles published in the last 20 years, (4) the sample size must be higher than 100 patients for SLE and RA studies and higher than 50 patients for SS, AIH, T1D, and MS studies, and (5) enough data available to calculate the prevalence of the antibodies in each AD. All of the search strategies included MeSH terms: “diabetes mellitus, type 1”, “lupus erythematosus, systemic”, “arthritis, rheumatoid”, “Sjögren's syndrome”, “hepatitis autoimmune”, and “multiple sclerosis”. In addition, key words for searching 20 antibodies were used including ANAs: antinuclear antibodies (ANAs), antidouble stranded DNA antibodies (Anti-dsDNA), antiribonucleoprotein antibodies (Anti-RNP), anti-Smith antibodies (Anti-Sm), Anti-Ro, Anti-La, lupic anticoagulant (LAC), IgG anticardiolipins, IgM anticardiolipins, anti-beta-2-glicoprotein I (Anti-*β*2GPI), rheumatoid factor (RF), anti-cyclic citrullinated peptide (Anti-CCP) antibodies, antiglutamic acid decarboxylase (Anti-GAD) antibodies, anti-islet cell antibodies (ICAs), anti-insulin antibodies (IAAs), antimitochondrial antibodies (AMAs), antismooth muscle antibodies (ASMAs), antithyroglobulin (Anti-TG) antibodies, and antithyroid peroxidase (Anti-TPO) antibodies. The complete search is described in detail in Table  1 in Supplementary Material available online at doi:10.1155/2012/569728.

## 3. Results

### 3.1. Meta-Analysis for Association between HLA-II Alleles and ADs

**Figure 2:**
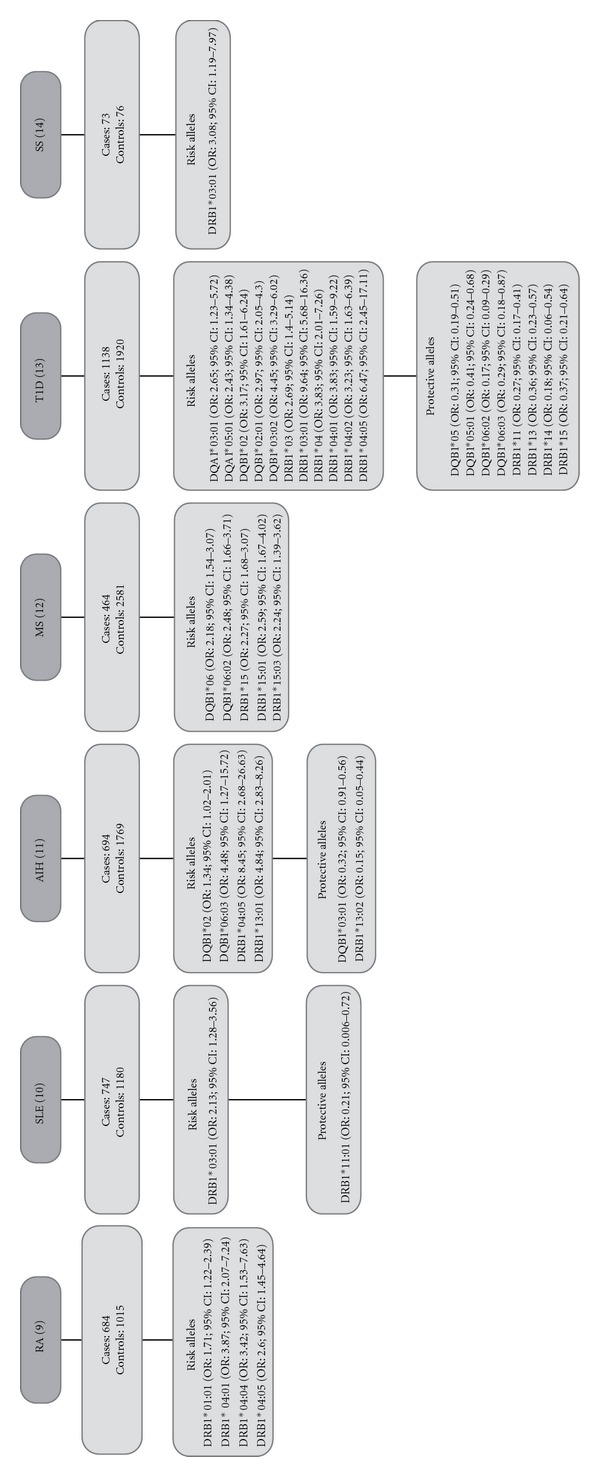
Previous results obtained from five meta-analyses and one original article.

A total of five meta-analysis of HLA class II polymorphisms in LA patients with ADs (RA, SLE, AIH, MS, and T1D) and the unique report for SS in LA were evaluated (Figure 2).

**Figure 3:**
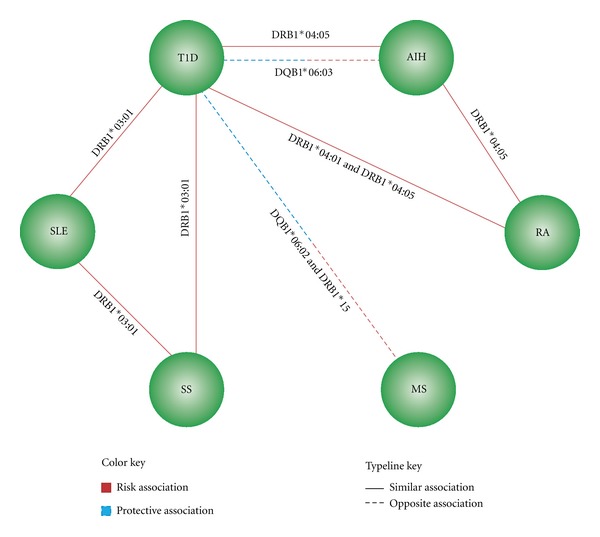
The complex interplay of HLA in six autoimmune diseases in Latin Americans.

A total of 3727 cases and 8465 controls were analyzed, and different types of association between alleles and ADs were found (Table 1). These included three risk alleles for two or more ADs, four opposite associations (the same allele is a risk factor for one AD, but a protective factor for other AD), thirteen risk alleles for a particular AD, and eight protective alleles that are disease-specific. The associations were grouped through network in Figure 3.

There are two risk alleles associated with three ADs. The first is DRB1*03:01 that was found to be a risk for SLE, SS, and T1D while the second is DRB1*04:05 that was associated with AIH, T1D, and RA. Similarly, there is one risk allele associated with two ADs. It is DRB1*04:01 which was found to impart risk for RA and T1D.

Interestingly, two opposite associations were found between MS and T1D. DQB1*06:02 and DRB1*15 alleles were risk factors for MS but protective factors for T1D. Likewise, an opposite association was found between AIH and T1D in that DQB1*06:03 was a risk factor for AIH but protective factor for T1D.

In addition, thirteen risk disease-specific alleles were found. Those are seven for T1D, three for MS, two for RA, and one for AIH while, conversely, eight protective alleles for a particular AD were reported. Those are five for T1D, two for AIH, and one for SLE (Table 1).

### 3.2. Study Quality

Significant heterogeneity was not seen for the DRB1*04:01 allele (*I* ^2^ = 0%; *Q* = 0; *P* = 0.98). Moderate heterogeneity for the DRB1*04:05 allele was observed (*I* ^2^ = 57%; *Q* = 4.65; *P* = 0.098). High heterogeneity was found by meta-analysis for the DRB1*03:01 allele (*I* ^2^ = 87.93%; *Q* = 16.57; *P* < 0.001). There was no evidence of publication bias in the current meta-analysis according to the Funnel plot and Egger's regression test (data not shown). 

### 3.3. Sharing of Autoantibodies in ADs

 Findings are summarized in Supplementary Table  2. Presence of ANAs was found in all of the analyzed ADs. These autoantibodies, as expected, were more prevalent in SLE (even over 75%) than other ADs. However, prevalence of ANAs over 60% was observed in SS, RA, and AIH. Anti-dsDNA, Anti-RNP and Anti-Sm antibodies were observed in SLE, RA, and SS. Anti-Ro and Anti-La antibodies were presented mainly in SS over 50%. Also, these two antibodies were presented in SLE, RA and AIH. In our revision, LAC was only present in SLE patients, but not in other ADs. IgG anticardiolipins were found in all ADs with different prevalences, SLE being the most frequent one. Otherwise, IgM anticardiolipins were presented in all ADs, but they were less prevalent than IgG subtype. Anti-*β*2GPI antibodies (IgG and IgM subtypes) were observed mainly in SLE, but they were present in all diseases, except in SS. RF was present in other ADs different to RA, such as SLE, SS, MS, and AIH. Likewise Anti-CCP antibodies were found in all ADs except in MS, although the prevalence was lower than 28%. Shared autoantibodies in ADs also were Anti-TPO and Anti-TG (present in all ADs except in AIH). Conversely, Anti-GAD, ICA, and IAA were observed only in T1D and AIH.

The prevalence of autoantibodies varied widely due to laboratory techniques, population, type of study, and activity of AD.

## 4. Discussion

In this meta-analysis, the genetic commonality in ADs was analyzed by examining the contributions from HLA-II alleles which confer associated risk or protection to six ADs: RA, SLE, AIH, MS, SS, and T1D in the LA population [9–14]. Two types of genetic risk factors were found: those common to many diseases and those specific to a given disorder. In addition, opposite associations between two different ADs and the same allele were found.

The LA population is a mixed group with ancestries that include blacks, Caucasians, and Amerindians, which reflects a notable racial, genetic, and cultural diversity [8]. However, our results showed that the effect of HLA-class II alleles on ADs in LA is similar to the reported effect on other populations regardless of latitudinal gradient and admixture. For instance, DRB1*03:01, DRB1*04:05, DRB1*04:01, and DQB1*02:01 risk alleles for T1D in LA also confer susceptibility in Caucasians and Asians [13]. DRB1*03:01 allele, which has been described in the Colombian population to be a risk factor for SS, was also associated with the disease at the worldwide level [54]. Furthermore, some non-HLA genes that influence the risk of developing ADs in Caucasians also have the same effect in Latin Americans (i.e., *C8orf13-BLK* and* CD226* genes) [55]. In contrast, some non-HLA genes influencing the developing ADs in a particular population are not replicated in another one (i.e., *PADI4* and *SLC22A4 *genes) [56].

Several studies have indicated that the major histocompatibility complex (MHC) is one of the central loci contributing to the development of ADs [25, 57]. Our results show that three alleles identified in previous analyses [9–14] of a particular disease were found to influence the risk of at least two diseases. The DRB1*03:01 allele was found to be a risk for SLE, SS, and T1D while DRB1*04:05 allele was associated with AIH, T1D, and RA. In addition, DRB1*04:01 allele confers susceptibility to T1D and RA. Analyses of other polymorphic genes related to autoimmune response and inflammation have been carried out. Results indicated that *PTPN22 *1858T/C [25] and *TNF-*α*-*308G/A [31, 58, 59] alleles are associated with SLE, SS, and T1D. Likewise, the *CTLA4* gene has been reported as a risk factor for AIH, T1D, and RA [37, 60, 61]. Other non-HLA genes that impart risk to develop two or more ADs in LA population have been also identified. For instance, *ITGAM* and its variant (rs1143679, Arg77His) are associated with SLE and systemic sclerosis (SSc) [62]. Another example is the association of rs6822844 in the IL2-IL21 region with SLE, T1D, and SS in non-European populations [24]. 

Our results demonstrated that there are both common susceptibility and protective alleles for ADs and single alleles involved in the development of ADs (Table 1). The DRB1*04:04 allele, which specifically influences susceptibility to acquire RA, was identified. It has a conserved motif (L-LE-[Q/R]-[R/K]-R-A-A) comprising residues 67–74 in the third hypervariable region of the DR*β*1 chain, known as the shared epitope (SE). These residues constitute an *α*-helical domain which forms one side of the antigen binding site, a site likely to affect antigen presentation [63]. Thus, the SE might selectively bind an arthritogenic peptide which could favor an autoimmune response. LA individuals carrying SE alleles have 3.5-fold higher risk of developing RA than noncarriers [9].

Although we identified common HLA class II alleles that contribute to susceptibility to different ADs, there is evidence indicating that two clinically distinct ADs with different susceptibility HLA-II alleles share other common genetics variants. Using a very large sample set, Zhernakova et al. compared the genetic basis of RA and celiac disease (CD). They found 14 loci that contribute to the risk of both diseases including *CD247*, *UBE2L3*, *DDX6*, *UBASH3A*, *SH2B3*, *8q24.2*, *STAT4*, and *TRAF1-C5*. However, it is known that RA and CD have different HLA risk alleles (HLA-DQ*A1 and DQ*B1 alleles in CD and HLA-DRB1 “SE” alleles in RA). According to the authors' hypothesis, the HLA-II molecules in these two ADs confer risk by preferentially presenting disease-specific antigens (gluten in CD, most likely citrullinated antigens in RA) to autoreactive T cells. Therefore, the disease specificity is determined in large part by the inheritance of specific HLA alleles and exposure to disease-specific antigens. The specific genes could be influencing downstream signaling events common to both diseases that may lead to altered T-cell activation and differentiation [64].

With regard to opposite associations DQB1*06:02 and DRB1*15 alleles were found to be risk factors for MS but protective factors for T1D. Our results are similar to those from other studies reporting that other MHC genes such as *CDSN* and *HLA-DMB* (rs3130981-A and rs151719-G, resp.) are risk factors for MS, but protective ones for T1D [45]. However, there is also evidence of the inverse relation. For instance, *TAP2* (rs10484565-T), *VARS2* (rs1264303-G), *NOTCH4* (rs2071286-A), *BTNL2* (rs2076530-G), and *TRIM40* (rs757262-T) were found to be risk factors for T1D but protective factors for MS [45]. Despite the presence of these genetically opposite associations, it is important to mention that clinical evidence supporting the coexistence of MS and T1D has been reported [65, 66]. Thus, these pleiotropic effects can be explained by the combined action of different alleles of several genes and environmental factors that change the biological context of the SNPs in different individuals and populations (Table 2).

Shared autoantibodies in ADs are described also. ANAs were presented in multiple ADs such as SLE, SS, RA, T1D, AIH, and MS. These autoantibodies are not specific for one AD. Furthermore, no autoantibody that was exclusive to a single AD was found. The theory that ADs have a common origin and similar pathogenic mechanisms receives support from these findings (Supplementary Table  2). These serological results reinforce the genetic findings of the present meta-analysis. In addition, there are pathophysiological mechanisms and clinical features supporting our findings (Table 2). There is evidence that an AD can be induced or triggered by infectious agents (i.e., viruses or bacteria) via different mechanisms, such as an alteration of expression of some genes involved in immune regulation, the induction of foreign proteins that could trigger the production of autoantibodies in B cells, and molecular mimicry [27]. Several epidemiologic studies have demonstrated that human endogenous retroviruses (HERVs), hepatitis C virus (HCV) [40], and Epstein-Barr virus (EBV) [43] are associated with different ADs (Table 2). Furthermore, elevated prevalence of HCV has been reported in ADs and suggests that it plays a pathogenic role triggering the production of ANAs, RF, anticardiolipin, and Anti-TG antibodies [40].

Another consideration concerning genetic findings is the familial aggregation. Relatives of patients with ADs have a higher risk for developing the same or other ADs than general population. These findings have been reported in AITD, RA, MS, SLE, and T1D [42, 47, 48].

Regarding the opposite association between AIH and T1D, there is one study with more than 250 AIH patients in which only two cases of T1D were presented [53]. Also, there is a report of one patient with AIH, T1D, and Grave′s disease (i.e., multiple autoimmune syndrome) [52]. Finally, one study in which children with AIH were evaluated for T1D-related autoantibodies and susceptibility alleles has been reported. da Silva et al. found a high prevalence of autoantibodies but despite these findings, the prevalence of risk alleles for T1D was similar to controls and only one patient developed T1D after 3 years [51].

In summary, our results validate the common origin of ADs paradigm. The finding of significant risk and protective alleles in LA and the fact that they are shared with other populations around the world highlights the primary role of some HLA regions in the genetic susceptibility to ADs regardless of latitudinal gradient and ethnicity.

## Supplementary Material

Supplementary Table 1: Detailed search of antibodies prevalence in the RA, SLE, AIH, T1D and SS
Supplementary Table 2: Prevalence of the the sharing of autoantibodies in RA, SLE, AIH, T1D and SS

## Figures and Tables

**Table 1:** Associations between HLA class II and six ADs: SLE, RA, T1D, AIH, SS, and MS.

Association	Allele	AD	OR	Lower limit	Upper limit	*P* value^a^
Risk (for only one AD)	DQA1*03:01	T1D	2.65	1.23	5.72	0.013
	DQA1*05:01	T1D	2.43	1.34	4.38	0.003
	DQB1*02:01	T1D	2.97	2.05	4.30	<0.001
	DQB1*03:02	T1D	4.45	3.29	6.02	<0.001
	DRB1*03	T1D	2.69	1.41	5.15	0.003
	DRB1*04	T1D	3.83	2.02	7.27	<0.001
	DRB1*04:02	T1D	3.23	1.63	6.39	0.001
	DQB1*06	MS	2.18	1.55	3.08	<0.001
	DRB1*15:01	MS	2.59	1.68	4.02	<0.001
	DRB1*15:03	MS	2.24	1.39	3.62	0.001
	DRB1*01:01	RA	1.71	1.23	2.39	0.002
	DRB1*04:04	RA	3.42	1.54	7.63	0.003
	DRB1*13:01	AIH	4.84	2.83	8.26	<0.001

Risk (for more than one AD)	DRB1*04:01	T1D and RA	3.86	2.32	6.42	<0.001
	DRB1*03:01	SLE, SS and T1D	3.56	1.42	11.54	0.009
	DRB1*04:05	AIH, T1D and RA	4.64	2.14	10.05	<0.001

Protection (for only one AD)	DQB1*05	T1D	0.31	0.19	0.51	<0.001
	DQB1*05:01	T1D	0.41	0.24	0.68	<0.001
	DRB1*11	T1D	0.27	0.17	0.42	<0.001
	DRB1*13	T1D	0.37	0.24	0.58	<0.001
	DRB1*14	T1D	0.18	0.06	0.55	0.002
	DQB1*03:01	AIH	0.33	0.19	0.56	<0.001
	DRB1*13:02	AIH	0.16	0.05	0.45	0.001
	DRB1∗11:01	SLE	0.21	0.006	0.72	<0.001

Opposite associations	DQB1*06:02	MS risk	2.49	1.67	3.71	<0.001
		T1D protection	0.17	0.09	0.29	<0.001
	DQB1*06:03	AIH risk	4.48	1.28	15.73	<0.001
		T1D protection	0.29	0.18	0.87	<0.001
	DRB1*15	MS risk	2.28	1.69	3.07	<0.001
		T1D protection	0.38	0.22	0.65	<0.001

^a^ *α* = 0.05.

Each OR and its CI show the effect size and precision for individual studies and for the combined effect calculated by the random model.

AD: autoimmune disease; RA: rheumatoid arthritis; SLE: systemic lupus erythematosus; AIH: autoimmune hepatitis; T1D: type 1 diabetes; SS: Sjögren's syndrome; MS: multiple sclerosis; OR: odds ratio.

**Table 2:** Relationship between genetic and clinical features with HLA-ADs associations.

Associations	Genetic associations (ref)	Clinical association (ref)
SLE, SS, T1D	Shared risk genes (i) *IL2–IL21 *(rs6822844) [24] (ii) *PTPN22 *(1858T/C) [25, 26] (iii) 8.1 Ancestral Haplotype [30] (iv) *TNF-*α** (-308G/A) [31–33]	Common clinical characteristics (i) Human endogenous retroviruses (HERVs) are associated with multiple ADs including SLE, SS, and T1D [27] (ii) Coexistence of SLE and SS has been reported [28, 29] (iii) Hepatitis C virus has been related to ADs such as RA, AIH, T1D, SLE, SS, and others [40] (iv) AIH was found in 0% to 1.7% of patients with SS. However, the prevalence of abnormal liver function test in SS patients is close to 47% [41] (v) High prevalence of ADs in siblings of probands affected by AITD, MS, RA, T1D, SLE, and others ADs [42]
AIH, RA, T1D	Shared risk genes (i) DRB1*04:05 [34–36] (ii) *CTLA4 *[37–39]	

MS, T1D	Shared risk genes	Common clinical characteristics
	(i) *CD226* (rs763361), *CLEC16A* (rs12708716), *SH2B3* (rs3184504) [43, 44] (ii) *ZSCAN23 *(rs11752919) [45]	(i) A latitudinal gradient characterizes both diseases. MS and T1D each become increasingly common as distance from the Equator increases [43]
	(iii)* KIF5A* (rs1678542), *SH2B3* (rs3184504), *CD226* (rs763361) [46]	(ii) Protective effect of vitamin D levels [43] (iii) Association to Epstein-Barr virus infection [43] (iv) Both MS and T1D are characterized by T cell-mediated autoimmunity. The targets of T cells are pancreatic islet and central nervous system antigens in both diseases [43] (v) Familial aggregation [47, 48]
	Shared protective genes: (i) HLA-DRB1*01, HLA-DRB1*10, HLA-DRB1*11, and HLA-DRB1*14 [43] Opposite gene associations: (i) Risk for T1D but protection for MS [45]:* TAP2* (rs10484565), *VARS2* (rs1264303), *CDSN* (rs1265048), *NOTCH4* (rs2071286), *BTNL2* (rs2076530), *TRIM40* (RS757262)	
	(ii) Risk for MS but protection for T1D [45, 49]: *CDSN* (rs3130981), *HLA-DMB *(rs151719) *IL2RA* (rs35285258), *IL2RA* (rs7090530)	

AIH, T1D	Shared protective alleles	Controversial characteristics
	(i) DQB1*03:01 [11, 50] Controversial genetic and clinical characteristics: (ii) In children with AIH, the frequency of high-risk HLA DQB1*03:02 or DQB1*02 alleles was low and similar to control frequencies, indicating low risk for DM despite the presence of DM-related autoimmunity markers [51]	(i) One case report with Grave's disease, AIH and T1D [52] (ii) One cohort of 278 patients with AIH presented only two cases of T1D [53] (iii) One study reported that the prevalence of ICA and IAA antibodies in children with AIH was 60.7 and 18.5% respectively. However, only one patient developed T1D [51]
